# Response of *Npt2a* knockout mice to dietary calcium and phosphorus

**DOI:** 10.1371/journal.pone.0176232

**Published:** 2017-04-27

**Authors:** Yuwen Li, Daniel Caballero, Julian Ponsetto, Alyssa Chen, Chuanlong Zhu, Jun Guo, Marie Demay, Harald Jüppner, Clemens Bergwitz

**Affiliations:** 1 Endocrine Unit, Massachusetts General Hospital and Harvard Medical School, Boston, Massachusetts, United States of America; 2 Department of Pediatrics, The First Affiliated Hospital, Nanjing Medical University, Nanjing, Jiangsu Province, China; 3 Section Endocrinology, Yale University School of Medicine, New Haven, Connecticut, United States of America; 4 Gastroenterology Unit, Massachusetts General Hospital and Harvard Medical School, Boston, Massachusetts, United States of America; 5 Department of Infectious Diseases, The First Affiliated Hospital, Nanjing Medical University, Nanjing, Jiangsu Province, China; 6 Pediatric Nephrology Unit, Massachusetts General Hospital and Harvard Medical School, Boston, Massachusetts, United States of America; The University of Tokyo, JAPAN

## Abstract

Mutations in the renal sodium-dependent phosphate co-transporters *NPT2a* and *NPT2c* have been reported in patients with renal stone disease and nephrocalcinosis, but the relative contribution of genotype, dietary calcium and phosphate to the formation of renal mineral deposits is unclear. We previously reported that renal calcium phosphate deposits persist and/or reappear in older *Npt2a*^*-/-*^ mice supplemented with phosphate despite resolution of hypercalciuria while no deposits are seen in wild-type (WT) mice on the same diet. Addition of calcium to their diets further increased calcium phosphate deposits in *Npt2a*^*-/-*^, but not WT mice. The response of PTH to dietary phosphate of *Npt2a*^*-/-*^ was blunted when compared to WT mice and the response of the urinary calcium x phosphorus product to the addition of calcium and phosphate to the diet of *Npt2a*^*-/-*^ was increased. These finding suggests that *Npt2a*^*-/-*^ mice respond differently to dietary phosphate when compared to WT mice. Further evaluation in the *Npt2a*^*-/-*^ cohort on different diets suggests that urinary calcium excretion, plasma phosphate and FGF23 levels appear to be positively correlated to renal mineral deposit formation while urine phosphate levels and the urine anion gap, an indirect measure of ammonia excretion, appear to be inversely correlated. Our observations in *Npt2a*^*-/-*^ mice, if confirmed in humans, may be relevant for the optimization of existing and the development of novel therapies to prevent nephrolithiasis and nephrocalcinosis in human carriers of *NPT2a* and *NPT2c* mutations.

## Introduction

Mutations in the sodium phosphate co-transporters, *NPT2a [1–3]* and *NPT2c* [4, 5] cause hypophosphatemic rickets with hypercalciuria (HHRH) and idiopathic hypercalciuria (IH). Affected individuals show renal phosphate-wasting, high circulating levels of 1,25(OH)_2_D and absorptive hypercalciuria. As a result they develop intraluminal stones (nephrolithiasis) and mineral deposits in the renal parenchyma (nephrocalcinosis) [4–7]. Furthermore, *NPT2a* has also been associated with nephrolithiasis [8] and altered renal function [9–11] in genome-wide association studies. Although little is known about the prevalence in stone patients, one compound heterozygous *NPT2a* mutations and one compound heterozygous carrier of *NPT2c* mutations was identified in a small cohort comprised of 272 genetically unresolved individuals (106 children and 166 adults) from 268 families with nephrolithiasis (n = 256) or isolated nephrocalcinosis (n = 16) [12]. Oral phosphate supplements are currently thought to reduce the risk for renal mineralization in carriers of *NPT2a* and *NPT2c* mutations by lowering circulating levels of 1,25(OH)_2_D and absorptive hypercalciuria. However, there is concern that, despite a reduction in urine calcium excretion, this therapy could contribute to the formation of renal calcium phosphate deposits under certain conditions.

This concern is based on several observations: i) renal calcium-phosphate deposits are found in the nephrocalcinosis that can develop in patients with X-linked hypophosphatemia (XLH) treated with oral phosphate supplements given multiple times throughout the day [13, 14] and in otherwise healthy individuals following treatment with phosphate enema [15] despite the absence of hypercalciuria; ii) in a recent survey of 27 kindreds with hereditary hypophosphatemic rickets with hypercalciuria (HHRH) we reported that a 10% decrease in tubular reabsorption of phosphate (TRP) predicts a two-fold increase in renal mineralization, independent of *NPT2c* mutation carrier status [16]; iii) dietary phosphate may increase the saturation product of calcium and phosphate by increasing urinary phosphate, which appears to be an important predictor of renal mineralization [17, 18]; iv) alterations in the levels of extracellular matrix factors affecting binding of phosphate to hydroxyapatite crystals such as *osteopontin (Opn)* or genes involved in the synthesis of pyrophosphate (PPi) and phosphate in the interstitial matrix such as *Extracellular nucleotide pyrophosphatase phosphodiesterase 1 (Enpp1)* are associated with renal mineralization [19, 20]. v) We recently reported that *Npt2a*^*-/-*^ mice show reduced urine osteopontin excretion when compared to WT mice and *Npt2a*^*-/-*^*;Opn*^*-/-*^ mice show an increased size of mineral deposits in their kidneys [21].

In the present study we compared the degree of renal mineralization of WT and *Npt2a*^*-/-*^ mice on diets with varying calcium and phosphate contents with the serum and urine biochemistries in response to these diets. Our findings suggest that *Npt2a*^*-/-*^ mice respond differently to dietary phosphate when compared to WT mice and that within the *Npt2a*^*-/-*^ cohort the degree of renal mineralization positively correlates with plasma phosphate and FGF23, and urinary calcium excretion, while it inversely correlates with urine phosphate and anion gap as a measure of proximal tubular bicarbonate and distal tubular ammonia excretion. Our observations in *Npt2a*^*-/-*^ mice, if confirmed in humans, may be relevant for the optimization of existing and the development of novel therapies to prevent nephrolithiasis and nephrocalcinosis in carriers of *NPT2a* and *NPT2c* mutations.

## Materials and methods

### Animals

Mice were euthanized in deep anesthesia with isoflurane by removal of vital organs. The research under IACUC protocol 2014–11635 was first approved Oct. 22 2014 by the Yale Institutional Animal Care and Use Committee (IACUC), renewed Sept. 7 2016, valid through Sept. 30 2017. Yale University has an approved Animal Welfare Assurance (#A3230-01) on file with the NIH Office of Laboratory Animal Welfare. The Assurance was approved May 5, 2015.

Male and female C57BL/6 mice were obtained from Charles River Laboratory, MA. Male and female *Npt2a*^*-/-*^ mice (B6.129S2-*Slc34a1*^*tm1Hten*^/J, Stock No: 004802), were purchased from The Jackson Laboratory (Bangor, ME). *Npt2c*^*-/-*^ mice were kindly provided by Dr. Hiroko Segawa, Dept. of Molecular Nutrition Institution of Health Bioscience, The Univ. of Tokushima Graduate School, Tokushima, Japan [22]. Mice were genotyped by PCR amplification of genomic DNA extracted from tail clippings and amplified by polymerase chain reaction (PCR) as described [22–25]. Mice were weaned at 3 weeks of age and allowed free access to water and normal chow (1.0% calcium, 0.7% phosphate, of which 0.3% is readily available for absorption, Harlan Teklad TD.2018S). At 8 weeks of age they were randomized to special diets using egg whites as protein source for 10 to 30 weeks: Normal phosphate, high calcium high vitamin D (TD.110762) contained 0.3% Pi, 2% Ca and 4.5 IU cholecalciferol (vitamin D_3_), phosphate deficient, high calcium, high vitamin D_3_ (TD.110761) contained 0.02% Pi, 2% Ca, 4.5 IU vitamin D_3_, HPC (high phosphate and high calcium) diet (TD.96348) contained 20% Lactose, 2.0% Ca, 1.25% Pi; HP (high phosphate) diet (TD.85349) contained 0.6% Ca, 1.2% Pi and CO (control diet) diet (TD.09803) contained 0.6%Ca, 0.3% Pi (S1 Fig). In all diets caloric content was 3.7 kcal/g, vitamin D_3_ content was 2 IU/g, the magnesium content was 0.2%. *Npt2a*^*-/-*^ mice can be maintained as homozygous line since they are viable and fertile. These mice were outbred against C57Bl6 wild-type mice and heterozygous mice were mated to obtain *Npt2a*^*-/-*^ and WT littermates to serve as controls with similar genetic background in our study. Since no differences were observed between genders data from males and females were pooled for the current study.

### Blood and urine parameters

Biochemical analyses were done on blood samples collected after cardiac puncture or orbital exsanguination following an overnight fast in deep anesthesia with isoflurane, immediately before animals were euthanized by removal of vital organs. Concentrations of serum and urinary total calcium (Ca), serum and urine inorganic phosphorus (S-P), urine sodium (U-Na), potassium (U-K), chloride (U-Cl) and blood urea nitrogen (S-BUN) were determined using Stanbio Laboratories (Boerne, Texas) kits #0155, #0830, #0140, #0160, #0210 and #0580, respectively. The concentration of urine creatinine (U-crea) and of serum 1,25-dihydroxyvitamin D (1,25(OH)_2_D) were determined using R&D systems (Minneapolis, MN) kit #KGE005 and #AC-62F1, respectively. Urine oxalate (U-oxalate) was determined using ABCAM kit #196990. Urine citrate was measured with the Roche Citric Acid UV-Method # 10139076035. Concentrations of plasma intact parathyroid hormone (PTH) and c-terminal fibroblast growth factor 23 (FGF23) were determined using Immutopics (San Clemente, CA) kit #60–2305 and #60–6300, respectively. c-terminal FGF23 ELISAs measure total FGF23 that includes intact FGF23 and its fragments. Unless altered processing of FGF23 is suspected total FGF23 ELISAs correlate well with the intact FGF23 ELISAs [26]. Internal standards were used to assure reproducibility between batches. The urine anion gap was calculated using the formula urine Na (mmol/l) + urine K (mmol/l)–urine Cl (mmol/l). SI correction factors are for Ca (mg/dl)*0.25 = Ca (mmol/L), P (mg/dl)*0.32 = P (mmol/L), creatinine (mg/dl)*88.4 = creatinine (umol/L). Fractional excretion indexes were calculated using the formula PEI = urine Pi/(urine creatinine*plasma Pi) or CEI = urine Ca/(urine creatinine*serum Ca), respectively.

### Inulin clearance

Inulin clearance was determined using serial tail bleedings following tail-vein injection of FITC-inulin as previously described [27]. Briefly, FITC-inulin (Sigma, St. Louis, MO) was dialyzed (molecular weight cutoff = 1,000) against 150 mM NaCl. 7.48μl/g body weight was injected via the tail vein. Tail vein blood was then collected at 5, 10, 20, 30, 40, 50, 60, 75, 90 and 120 min post injection of FITC-inulin and the plasma was assessed for FITC fluorescence (λ excitation = 485 nm; λ emission = 535 nm) using a Victor3 plate reader (PerkinElmer, Waltham, MA). Mice tolerated serial tail bleeds well permitting us to measure the same mice after 10, 20 and 30 weeks on HPC diet. GFR was calculated by fitting the data to a biexponential decay function and using the equation GFR = I/(A/α)+B/β), where I is the amount of FITC-inulin delivered by the bolus injection, A and B are the y-intercepts of the two decay components, and α and β are the corresponding decay constants for the distribution and elimination phases, respectively [28].

### Kidney histology

Left kidneys were fixed in 4% formalin/PBS at 4°C for 12 h and then dehydrated with increasing concentration of ethanol and xylene, followed by paraffin embedding. Mineral deposits were determined on 10 um von Kossa stained sections counterstained with 1% methyl green. Hematoxyline/eosin was used as counterstain for morphological evaluation. Histomorphometric evaluation was performed using an Osteomeasure System (Osteometrics, Atlanta, GA). % calcified area was determined using the formula: calc. area = 100*calcified area/total area, and mineralization size was determined using the formula: calc. size = calcified area/number of mineralization.

For transmission electron microscopy a 1 mm^3^ block of the left kidney was fixed in 2.5% Glutaraldehyde and 2% paraformaldehyde in phosphate buffered saline for 2 hrs., followed by post-fixation in 1% osmium liquid for 2 hours. Dehydration was carried out using a series of ethanol concentrations (50% to 100%). Renal tissue was embedded in epoxy resin, and polymerization was carried out at 60°C for overnight. After preparing a thin section (50 nm), the tissues were double stained with uranium and lead and observed using a Tecnai Biotwin (LaB6, 80 kV) (FEI, Thermo Fisher, Hillsboro, OR) at the Yale Center for Cellular and Molecular Imaging (YCCMI).

### Statistical analysis

Data are expressed as means±SEM and were analyzed in Prism 7.0 (GraphPad Software, Inc., La Jolla, CA) and JMP Pro 11 (SAS, Cary, NC). Differences between groups were considered significant, if p-values obtained with linear regression analysis, or with two-way ANOVA were smaller than 0.05. Tukey’s test for multiple comparisons was used where indicated.

## Results

### *Npt2a*^*-/-*^ mice form renal mineral deposits on HP diet

**Fig 1:**
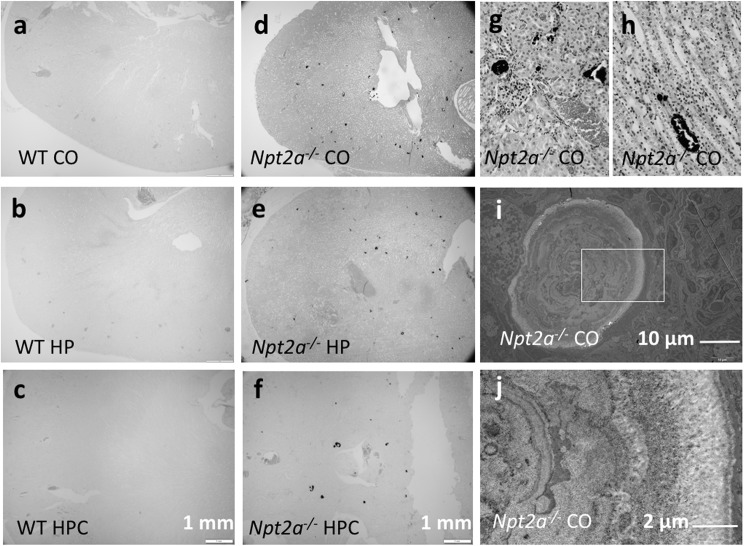
Cortical and medulary renal mineralization. Light micrographs of 10 um renal sections, prepared from paraffin-embedded kidneys, of mice were fed different diets. WT (**a-c**), *Npt2a*^*-/-*^ (**d-f**), von Kossa&methylene green, 4X; *Npt2a*^*-/-*^ on CO diet, renal cortex (**g**) and medulla (**h**), von Kossa&hematoxin&eosin, 40X. Transmission electron micrographs showing microspheres in *Npt2a*^*-/-*^ on CO diet (**i**), inset with larger magnification shown in (**j**).

Humans with loss-of-function of *NPT2a [1–3]* and *NPT2c* [4, 5] develop renal mineralization, which may manifest during early childhood prior to specific therapy or when inappropriately receiving active vitamin D analogs, but can also occur later in life [6]. To model these kidney abnormalities, we initially tested *Npt2a*^*-/-*^ and *Npt2c*^*-/-*^ mice [22, 24]. Diets with standard calcium and phosphate content (Ca 1.0%, Pi 0.6%) were not reported to induce renal mineralization beyond weaning age in *Npt2a*^*-/-*^ mice [29] and no mineralization was reported in *Npt2c*^*-/-*^ mice up to 12 weeks of age [22]. We therefore first tested the effect of a phosphate deficient (Pi 0.02%), high calcium (Ca 2.0%) and high vitamin D_3_ (4.5 IU/g) diet intended to maximize hypercalciuria in both mouse models as reported for human individuals with HHRH [6] and mice [30]. However, no renal mineralization was observed at birth, at weaning and up to 12 weeks of life in either mouse strain. Conversely, renal mineralization was seen in *Npt2a*^*-/-*^ mice when the phosphate content of these diets was raised to 0.3% in CO diet (Fig 1D), while still no such changes were observed in *Npt2c*^*-/-*^ mice (not shown). Renal mineralization was present in both intraluminal and interstitial compartments (Fig 1G and 1H), and in addition to staining with the phosphate dye, von Kossa deposits were also positive with the calcium dye alizarin red (not shown). Furthermore, transmission electron images showed concentric calcium phosphate spheres similar to those described by others [29, 31] (Fig 1I and 1J).

Taken together, these findings suggest that dietary phosphate supports the formation of renal mineral deposits, at least under certain conditions, which is contrary to the current belief that oral phosphate supplementation reduces risk for renal calcification in phosphate wasting disorders by normalizing urine calcium excretion.

To further evaluate the dietary conditions influencing the development of renal mineralization, we placed 2-month-old *Npt2a*^*-/-*^ and wild-type (WT) littermates on three diets containing differing amounts of calcium and phosphate for 10 weeks, while the nutritional vitamin D and magnesium content were kept unchanged: i) HPC diet (High phosphate and calcium diet; 20% lactate, 2% calcium, 1.25% phosphate); ii) HP diet (High phosphate diet; 0.6% calcium, 1.20% phosphate); or iii) CO diet (Control diet; 0.6% calcium, 0.3% phosphate)(S1 Fig). Lactate in the first diet was shown to increase intestinal absorption of calcium [32]. Size and body weight (BW) of mice in each diet group were indistinguishable and the animals appeared to be thriving well, suggesting that intake of these diets was comparable.

### Serum and urine biochemistry of *Npt2a*^*-/-*^ mice on diets with different calcium and phosphate contents compared to WT

**Fig 2:**
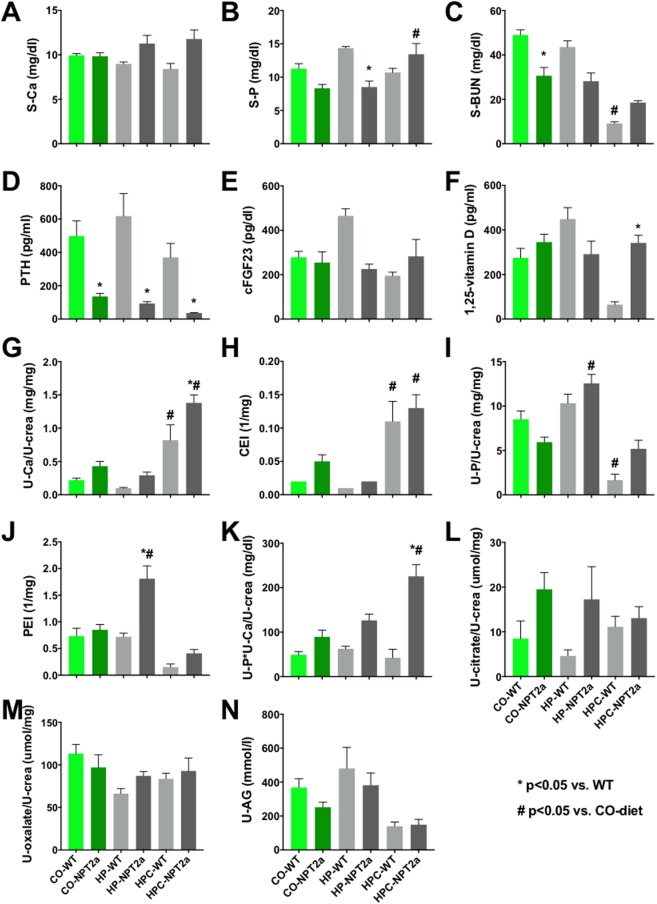
Biochemical parameters. Serum phosphorus (S-P), serum calcium (S-Ca), serum 1,25(OH)_2_-vitamin D (1,25-D), plasma intact PTH (PTH), plasma c-terminal FGF23 (cFGF23), serum blood urea nitrogen (S-BUN), phosphate excretion index (U-Pi/(S-Pi*u-creatinine)(PEI), calcium excretion index (U-Ca/(S-Ca*U-creatinine) (CEI), citrate (U-citrate), oxalate (U-oxalate) and anion gap (U-AG). 8 weeks old mice were placed for 10 weeks on special egg-white based diets: HPC diet (High phosphate and calcium diet; 20% lactate, 2% calcium, 1.25% phosphate); HP diet (High phosphate diet; 0.6% calcium, 1.20% phosphate); CO diet (Control diet; 0.6% calcium, 0.3% phosphate); WT: wild type; Npt2a: *Npt2a*^*-/-*^ mice. The data represent mean±SEM; p-values were obtained by ANOVA and Tukey’s test to correct for multiple comparison, selected comparisons shown here, see complete list of p-values in S1 Table.

Consistent with previous reports [24, 29] when compared to WT mice serum Pi, plasma PTH and FGF23 were decreased in *Npt2a*^*-/-*^ mice on the CO diet, while serum 1,25(OH)_2_D and urine calcium were increased (Fig 2, Table 1 and S1 Table), albeit only plasma PTH remained significantly decreased after Tukey’s correction for multiple comparisons. HP diet increased phosphaturia in *Npt2a*^*-/-*^ mice and HPC diet increased calciuria in WT and *Npt2a*^*-/-*^ mice. The urine calcium phosphorus product was increased in *Npt2a*^*-/-*^ mice on all three diets but not in WT mice, albeit significantly only on HPC diet. Lack of increase of the excretion of phosphate on HPC diet when compared to HP diet may be due to decreased intestinal phosphate absorption as CaHPO_4_ salt and suppression of PTH by this diet’s calcium content.

**Table 1:** Two-way ANOVA analysis.

	Diet	genotype
**S-Ca**	0.51	**0.04**
**S-P**	**0.03**	**0.01**
**S-BUN**	**<0.0001**	**0.01**
**PTH**	**0.05**	**<0.0001**
**cFGF23**	0.57	0.28
**1,25-D**	0.20	0.10
**U-Ca/U-crea**	**<0.0001**	**0.0003**
**CEI**	**<0.0001**	**0.04**
**U-P/U-crea**	**<0.0001**	0.46
**PEI**	**<0.0001**	**0.01**
U-P*U-Ca/U-crea	**0.0002**	**<0.0001**
**U-Citrate/U-crea**	0.82	**0.04**
**U-oxalate/U-crea**	0.12	0.76
**U-AG**	**0.004**	0.19

The two genotypes and three diet groups from Fig 2 were subjected to a two-way ANOVA, illustrating that Npt2a-/- mice respond differently to their diets. The number of animals included for each diet is shown in S1 Table.

**Fig 3:**
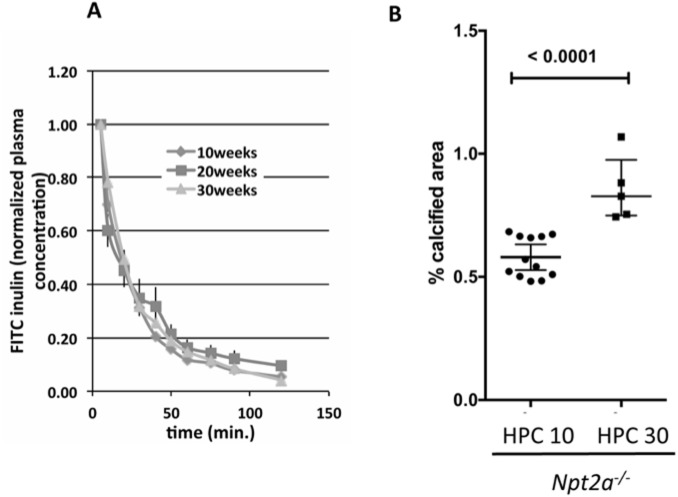
Inulin-clearance is stable in *Npt2a*^*-/-*^ mice on HPC diet for 10, 20 and 30 weeks despite increased renal mineralization. **(A)** Inulin-clearance is stable in *Npt2a*^*-/-*^ mice placed at 8 weeks of age on HPC diet for 10, 20, and 30 weeks, and 175±8, 180±9, and 165±6 ml/min., respectively (mean±SEM) (**B)** Renal mineralization continues to increase on HPC diet over time from 10 weeks (HPC10) to 30 weeks (HPC30). The data represent individual animals (closed circles) with the means±SEM, p-values shown above the lines of comparisons were calculated by Student’s t-test.

Serum BUN levels were in the normal range for all groups, but lower in mutant mice on CO diet and in WT mice on HPC diet. Inulin-clearances measured in the same *Npt2a*^*-/-*^ mice on HPC diet for 10, 20 and 30 weeks were unaffected (Fig 3A) despite progressive renal mineralization (Fig 3B).

Since urine pH affects renal mineralization, we also determined urine anion gap, which indirectly measures urinary ammonia excretion [33–35]. However, no difference between genotypes and diets was observed. Likewise, no differences were seen for urine excretion oxalate and citrate.

Two-way ANOVA analysis (Table 1) showed a significant effect of diet for S-P, S-BUN, PTH, U-Ca/U-crea, CEI, U-P/U-crea, PEI, U-P*U-Ca/U-crea and U-AG, while there was a significant effect of genotype on S-Ca, S-P, S-BUN, PTH, U-Ca/U-crea, CEI, PEI, U-P*U-Ca/U-crea, and U-Citrate/U-crea. Collectively, these finding suggests that *Npt2a*^*-/-*^ mice respond differently to dietary phosphate when compared to WT mice.

### Addition of calcium to their diet further increased calcium phosphate deposits in *Npt2a*^*-/-*^, but not in WT mice

**Fig 4:**
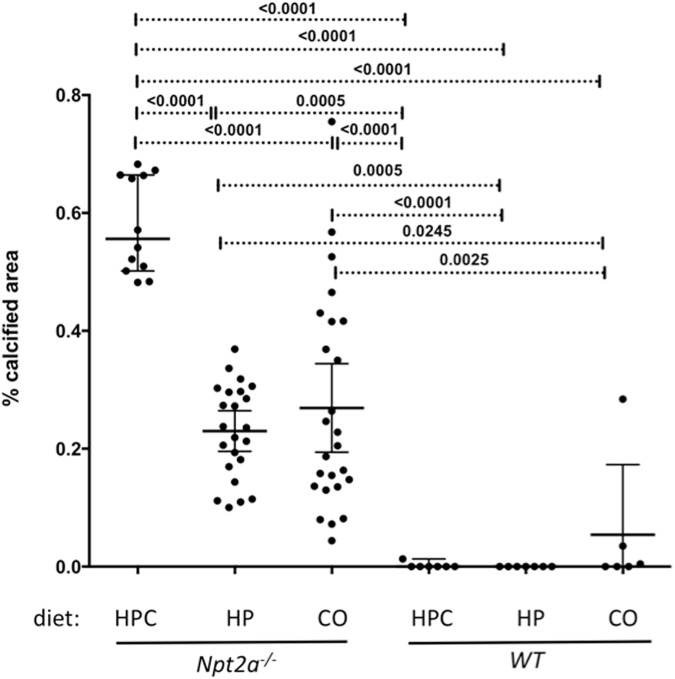
Renal mineralization is increased in *Npt2a*^*-/-*^ mice on high phosphate/high calcium diet. Histomorphometric analysis of renal mineralization (%calcified area = 100*mineralization area/tissue area) in 10 um sections of kidneys from mice feed different diets for 10 weeks (see S1 Fig for layout and legend of Table 2 for composition of diets). The data represent individual animals (closed circles) and the mean±SEM; p-values were obtained by ANOVA and Tukey’s test to correct for multiple comparison.

Following 10 weeks on the respective diets the animals were sacrificed, kidneys of *Npt2a*^*-/-*^ mice fed HPC diet (n = 12) showed 0.58±0.08% calcified area, while *Npt2a*^*-/-*^ mice fed CO diet (n = 21) showed 0.27±0.18% calcified area (p<0.0001 vs. HPC diet) (Fig 4). Mineralized area was reduced in *Npt2a*^*-/-*^ mice fed a HP diet (0.23±0.08% calcified area, n = 23) when compared to HPC diet, but was similar when compared to *Npt2a*^*-/-*^ mice fed CO diet. No mineralization was observed in WT mice on HPC or HP diet, but mineralization was seen in two of ten WT mice on CO diet, albeit less than in *Npt2a*^*-/-*^ mice on the same diet. Mineralization size was similar on all three diets (calculation see methods, data not shown).

### Degree of renal mineralization of *Npt2a*^*-/-*^ mice directly correlates with blood phosphate and FGF23 levels and urine calcium excretion

A combined univariate linear regression analysis of all *Npt2a*^*-/-*^ mice showed a significant direct correlation of the urine calcium/urine creatinine ratio (U-Ca/U-cre, CI = 0.49, p = 8.14E-05, n = 59) (Table 2, S1G Fig) and of the calcium excretion index (CEI, CI = 0.39, p = 0.00259, n = 57) (Table 2, S2H Fig) with % calcified area. This analysis also showed a positive correlation of serum phosphate (S-P, CI = 0.39, p = 0.00402, n = 55) (Table 2, S2B Fig) and plasma FGF23 with % calcified area (cFGF23, CI = 0.36, p = 0.01036, n = 49) (Table 2, S2E Fig).

**Table 2:** Linear regression analysis.

	Univariate analysis			Multivariate analysis								
Parameter	CC	p-values	n	Sex	S-P	cFGF23	U-Ca/U-crea	CEI	U-P/U-crea	PEI	U-AG	Multiple
**S-Ca**	0.12	0.4	60	**<0.0001**	1.0	0.6	**0.0001**	**0.0009**	**0.03**	**0.02**	0.5	0.3
**S-P**	0.39	**0.004**	55	**0.006**	**0.004**	**0.009**	0.1	0.1	**0.01**	0.1	**0.01**	1.0
**S-BUN**	0.07	0.6	59	0.3	**0.03**	0.7	0.1	0.1	0.68	0.2	0.2	1.0
**PTH**	0.05	0.7	48	**0.0003**	0.3	0.3	**0.03**	0.1	1.0	0.8	0.4	0.4
**cFGF23**	0.36	**0.01**	49	**0.01**	**0.004**	**0.01**	**0.009**	**0.003**	**0.03**	**0.03**	0.2	0.2
**1,25-D**	-0.01	1.0	46	0.7	0.9	0.7	0.7	0.8	0.5	0.5	0.2	0.7
**U-Ca/U-crea**	0.49	**<0.0001**	59	**<0.0001**	**0.004**	**0.001**	**<0.0001**	**0.009**	**0.0008**	**0.002**	**0.002**	0.8
**CEI**	0.39	**0.003**	57	**0.0002**	**0.03**	**0.01**	0.4	**0.002**	**0.01**	**0.03**	**0.008**	0.4
**U-P/U-crea**	-0.27	**0.04**	59	**0.04**	**0.04**	0.1	0.4	0.23	**0.04**	0.81	0.6	0.4
**PEI**	-0.37	**0.008**	53	**0.01**	0.2	**0.003**	0.1	0.08	0.2	**0.008**	0.3	0.4
U-P*U-Ca/U-crea	0.23	0.09	57	0.2	0.7	0.2	0.1	0.7	**0.02**	0.09	**0.01**	**0.02**
**U-Citrate/U-crea**	0.15	0.4	32	0.4	0.5	0.5	0.2	0.2	0.3	0.3	0.94	0.6
**U-oxalate/U-crea**	0.20	0.3	33	0.4	0.34	0.6	0.2	0.07	0.09	0.1	0.3	0.1
**U-AG**	-0.42	**0.01**	35	**0.02**	**0.008**	0.1	0.06	**0.04**	**0.03**	**0.008**	**0.01**	**0.001**

Following univariate linear regression analysis of all experimental *Npt2a*^*-/-*^ mice analysis of covariance (multivariate analysis) was used to control for influence of gender, variables separately as indicated in the column header or to control for multiple variables (S-P, cFGF23, U-Ca/U-crea, CEI, U-P/U-crea, PEI, U-AG). The number of animals included for each diet is shown in S1 Table.

### Degree of renal mineralization of *Npt2a*^*-/-*^ mice inversely correlates with urine phosphate excretion and urine anion gap

Univariate linear regression analysis furthermore indicated a significant inverse correlation of the urine phosphate/urine creatinine ratio (U-P/U-cre, CI = -0.27, p = 0.03855, n = 59) (Table 2, S3A Fig) and of the phosphate excretion index (PEI, CI = -0.37, p = 0.0084, n = 53) (Table 2, S3B Fig) with % calcified area. Urine anion gap was inversely related to the degree of renal mineral deposits (U-AG, CI = -0.42, p = 0.01271, n = 35) (Table 2, S3F Fig). No significant association was seen for PTH in Table 2 and S2D Fig although comparison of the means in Fig 2D and S1 Table suggests an inverse relationship between PTH levels and mineralization. Likewise, no significant association was observed for urine citrate/urine creatinine, urine oxalate/urine creatinine or serum 1,25(OH)_2_D. Similar trends were seen when evaluating diet groups separately (not shown).

### Multivariate linear regression analysis suggests that plasma phosphate, serum FGF23, urine calcium, urine phosphate and anion gap are independent predictors of renal mineral deposits

The observed associations continued to be significant after controlling for gender, or the respective variables independently (Table 2). Urine anion gap remained significant even when controlling for all significant variables simultaneously. A stepwise multivariate linear regression analysis furthermore showed that plasma phosphate was able to explain 58% of the variance in renal mineralization, and both plasma phosphate and CEI combined were able to explain 69% of the variance.

## Discussion

Oral phosphate supplements are currently thought to reduce risk for renal mineralization in human carriers of *NPT2a* and *NPT2c* mutations. However, as mentioned in the introduction, there is concern that this therapy might contribute to the formation of renal mineralization despite reduced 1,25(OH)_2_D levels and thus reduced urinary calcium excretion under certain conditions. Our observation that no mineralization was observed in *Npt2a*^*-/-*^ and *Npt2c*^*-/-*^ mice on phosphate deficient diet, while mineralization persisted and/or reappeared in older *Npt2a*^*-/-*^ mice supplemented with 0.3% phosphorus, further supports this concern.

Tenenhouse et al. [30] found that renal mineralization in *Npt2a*^*-/-*^ mice resolves at weaning age when the dietary phosphate content was increased from 0.6% to 1%. However, these authors also noticed that mineralization re-appeared when phosphate was further raised to 1.65% despite improved hypercalciuria on this diet. Similarly, we found continued mineralization in older *Npt2a*^*-/-*^ mice on HP diet containing 1.2% Pi, despite low calciuria, when compared to CO diet and HPC diet (Figs 2 and 4), suggesting that dietary phosphate can be harmful under certain conditions, and that oral phosphate supplementation to treat the bone disease in hypophosphatemic rickets may need to be carefully monitored to not cause renal calcifications.

Renal calcifications were similar when phosphate content was raised from 0.3 to 1.2% in HP diet and only addition of 2% calcium in HPC diet made them worse, suggesting that dietary calcium or the ratio of dietary calcium and phosphorus contributes to mineralization risk.

Renal mineralization was absent in WT mice on HPC and HP diets. These observations suggest that *Npt2a*^*-/-*^ mice respond differently to dietary phosphate and calcium supplementation when compared to WT.

To better understand the impact of dietary phosphate we considered the possibility that dietary phosphate increases the risk for renal mineralization by raising urine phosphate or the urine calcium x phosphorus product. *Npt2a*^*-/-*^ mice are predicted to be more susceptible to negative effects of dietary phosphate due to their reduced ability to reclaim phosphate from the urine when compared to WT mice. This hypothesis is supported by the finding that phosphaturia and calcium x phosphorus product (U-Ca*U-P/U-crea) is higher in *Npt2a*^*-/-*^ mice on HP diet when compared to WT (Fig 2I–2K and S1 Table).

Linear regression analysis of the serum and urine biochemistries of *Npt2a*^*-/-*^ mice revealed a positive correlation between plasma phosphorus and % calcified area (S2B Fig) and plasma FGF23 and % calcified area (S2E Fig), further supporting the idea that dietary phosphate, by increasing plasma phosphorus and FGF23, can worsen renal calcifications.

It is possible, that FGF23 directly supports renal calcifications in addition to being a marker for oral phosphate load and FGF23-neutralizing antibodies, which have successfully been used in XLH [36] may offer advantages for the risk of renal calcifications when compared to standard therapy with oral phosphate supplements.

However, in light of the positive correlation of plasma phosphate and FGF23 with renal mineralization, we were surprised to find phosphaturia (U-P/U-crea and PEI) inversely related to renal mineralization (Table 2 and S3A and S3B Fig). Furthermore, renal mineralization was present in both intraluminal and interstitial compartments (Fig 1G and 1H), while loss of *Npt2a* by modifying reabsorption of phosphate from the urine would be predicted to cause nephrocalcinosis rather than nephrolithiasis in these mice [17, 18]. Thus additional factors may determine risk for renal mineralization in addition to increased intraluminal phosphate in *Npt2a*^*-/-*^ mice on HP diet, for example reduced osteopontin excretion as previously reported by us [21], or interstitial levels of phosphate.

We also evaluated for changes in other stone risk factors [17, 18], but no differences were observed in urine citrate and oxalic acid excretion when comparing WT and *Npt2a*^*-/-*^ mice (Fig 2L and 2M, S1 Table), or when using linear regression analysis of the *Npt2a*^*-/-*^ cohort (Table 2, S3F and S3G Fig). However, urine anion gap, which is an indirect measure of renal ammonia excretion, was found to be inversely correlated with the degree of renal mineralization (Table 2, S3H Fig). High urine anion gap is characteristically seen with impaired urine ammonia excretion in renal tubular acidosis type 1, while low or negative urine anion gap can occur in the context of proximal tubular bicarbonate loss in renal tubular acidosis type 2 [33–35]. The latter would be consistent with reports of Fanconi-type syndrome due to loss-of-function mutations in *NPT2a* in human individuals [2], and suggests that urine pH could be an additional risk factor for stone formation in *Npt2a*^*-/-*^ mice.

The observed inverse relation of urine anion gap with the degree of renal mineralization persisted when controlled for P-P, cFGF23, U-Ca/U-crea, CEI, U-P/U-crea, PEI, U-AG separately or in combination (Table 2), and likewise when *Npt2a*^*-/-*^ mice were analyzed for each diet separately (not shown). Taken together our findings suggest that proximal tubular function beyond phosphate transport may be impaired which could contribute to the formation of renal mineralization in *Npt2a*^*-/-*^ mice.

A limitation of this study is that *Npt2a*^*-/-*^ mice exhibit a milder biochemical phenotype than that seen in most humans with loss-of-function mutations in *NPT2a*. Renal mineralization also resolves after weaning [29, 31] in this mouse model and composition of mineral deposits may differ between mice and humans who carry *NPT2a* mutations. However, our findings that renal stones and nephrocalcinosis persist and/or reappear in older *Npt2a*^*-/-*^ mice under certain conditions, and earlier reports and our own TEM studies show that these mineral deposits have a composition similar to Randall’s plaques ([31] and Fig 1I and 1J) argue that despite these species-related differences, important insights can be gained into the underlying pathophysiology of nephrolithiasis and nephrocalcinosis in this mouse model. Metabolic cage studies are needed to formally assure similar intake of the different diets, however similar weight gain of all three cohorts on CO, HP and HPC diets is reassuring. Direct determination of urine pH and bicarbonate excretion requires dissection of bladders from a new cohort of mice and will be subject of future studies to confirm indirect evidence obtained from urine anion gap measurements presented here. Lastly, a time course of renal mineralization is necessary to determine whether neonatal and weaning-age mineralization, which presumably formed during relatively high calcium and phosphate intake with the breast milk, did not resolve when animals are maintained on HP diet after weaning. Or whether new mineralization developed on HP diet, as suggested by our observation that renal mineralization continues to increase in mice between 10 and 30 weeks on HPC diet (Fig 3B).

In summary, we show here that *Npt2a*^*-/-*^ mice respond differently to dietary phosphate when compared to WT mice and that the degree of renal mineralization positively correlates with serum phosphate, plasma FGF23, and urinary calcium excretion, while it inversely correlates with urine phosphate and urine anion gap. Our observations in *Npt2a*^*-/-*^ mice suggest presence of risk factors for renal mineralization in addition to hypercalciuria, and if confirmed in humans our findings may be relevant for the optimization of existing and for the development of novel therapies to prevent nephrolithiasis and nephrocalcinosis in human carriers of *NPT2a* and *NPT2c* mutations.

## Supporting information

S1 Fig Experimental design. At 8 weeks of age they were randomized to special diets for 10 to 30 weeks: HPC (high phosphate and high calcium diet, 20% Lactose, 2.0% Ca, 1.25% Pi; HP (high phosphate diet, 0.6% Ca, 1.2% Pi and CO (control diet, 0.6%Ca, 0.3% Pi). Mice were sacrificed at after 10 weeks or 30 weeks on these diets.
(TIF)

S2 Fig Urinary calcium excretion and plasma FGF23 levels are positively correlated with renal mineralization in a combined univariate linear regression analysis *Npt2a*^-/-^ mice fed different diets. All experimental *Npt2a*^*-/-*^ mice from S1 Table (n = 56) were evaluated using linear regression analysis to determine the association of renal mineralization with serum calcium (S-Ca, **A**), serum phosphorus (S-P, **B**), serum BUN (S-BUN, **C**), plasma intact PTH (PTH, **D**), plasma c-terminal FGF23 (cFGF23, **E**), serum 1,25(OH)_2_-vitamin D (1,25(OH)_2_-D, **F**), the ratios of urine calcium/urine creatinine (U-Ca/U-crea, **G**), and urine calcium excretion index (CEI, **H**). Data points represent values of individual animals. Results of the linear regression analysis are shown as solid line with 95% confidence interval (stippled lines), for correlation coefficients and Pearson’s p-values see Table 2.
(TIFF)

S3 Fig Urinary phosphate excretion and anion gap are negatively correlated with renal mineralization in a combined univariate linear regression analysis *Npt2a*^-/-^ mice fed different diets. All experimental *Npt2a*^*-/-*^ mice from S1 Table (n = 56) were evaluated using linear regression analysis to determine the association of renal mineralization with the ratios of urine phosphorus/urine creatinine (U-P/U-crea, **A**), urine phosphate excretion index (PEI, **B**), urine calcium*phosphorus/urine creatinine (U-Ca*U-P/U-crea, **C**), urine citrate/urine creatinine (U-citrate/U-crea, **D**), urine oxalate/urine creatinine (U-oxalate/U-crea, **E**), urine anion gap (U-AG, **F**). Data points represent values of individual animals. Results of the linear regression analysis are shown as solid line with 95% confidence interval (stippled lines), for correlation coefficients and Pearson’s p-values see Table 2.
(TIFF)

S1 Table Biochemical parameters. Serum phosphorus (S-P), serum calcium (S-Ca), serum 1,25(OH)_2_-vitamin D (1,25-D), plasma intact PTH (iPTH), plasma c-terminal FGF23 (cFGF23), serum blood urea nitrogen (S-BUN), phosphate excretion index (U-Pi/(S-Pi*u-creatinine)(PEI), calcium excretion index (U-Ca/(S-Ca*U-creatinine) (CEI), citrate (U-citrate), oxalate (U-oxalate) and anion gap (U-AG). All diets are egg-white based: HPC diet (High phosphate and calcium diet; 20% lactate, 2% calcium, 1.25% phosphate); HP diet (High phosphate diet; 0.6% calcium, 1.20% phosphate); CO diet (Control diet; 0.6% calcium, 0.3% phosphate); WT, wild type; The data represent mean±SEM; p-values were obtained by ANOVA and Tukey’s test to correct for multiple comparison.
(XLSX)
