# Supplementary figures and images for: Response of Npt2a knockout mice to dietary calcium and phosphorus
Source: PLoS One. 2017 Apr 27;12(4):e0176232. doi: 10.1371/journal.pone.0176232 (PMC5407772; doi:10.1371/journal.pone.0176232)

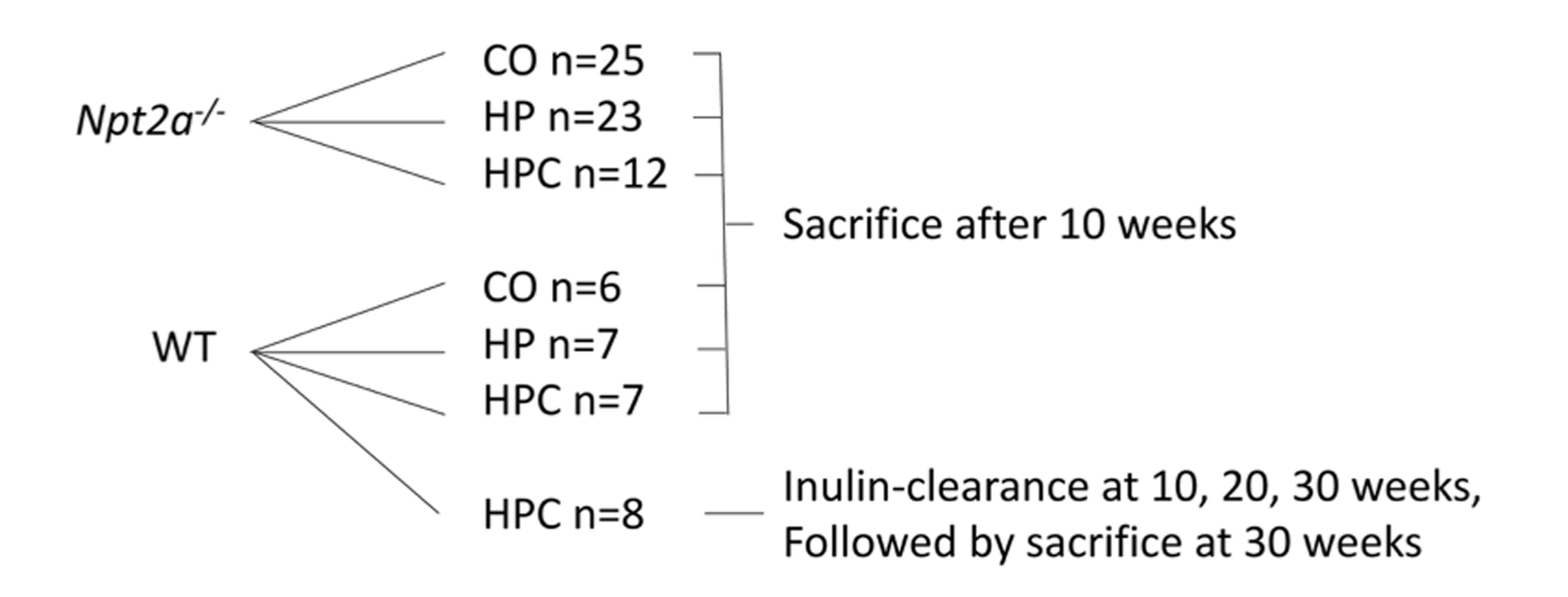

Supplement: S1 Fig — At 8 weeks of age they were randomized to special diets for 10 to 30 weeks: HPC (high phosphate and high calcium diet, 20% Lactose, 2.0% Ca, 1.25% Pi; HP (high phosphate diet, 0.6% Ca, 1.2% Pi and CO (control diet, 0.6%Ca, 0.3% Pi). Mice were sacrificed at after 10 weeks or 30 weeks on these diets. (TIF) [file pone.0176232.s001.tif]

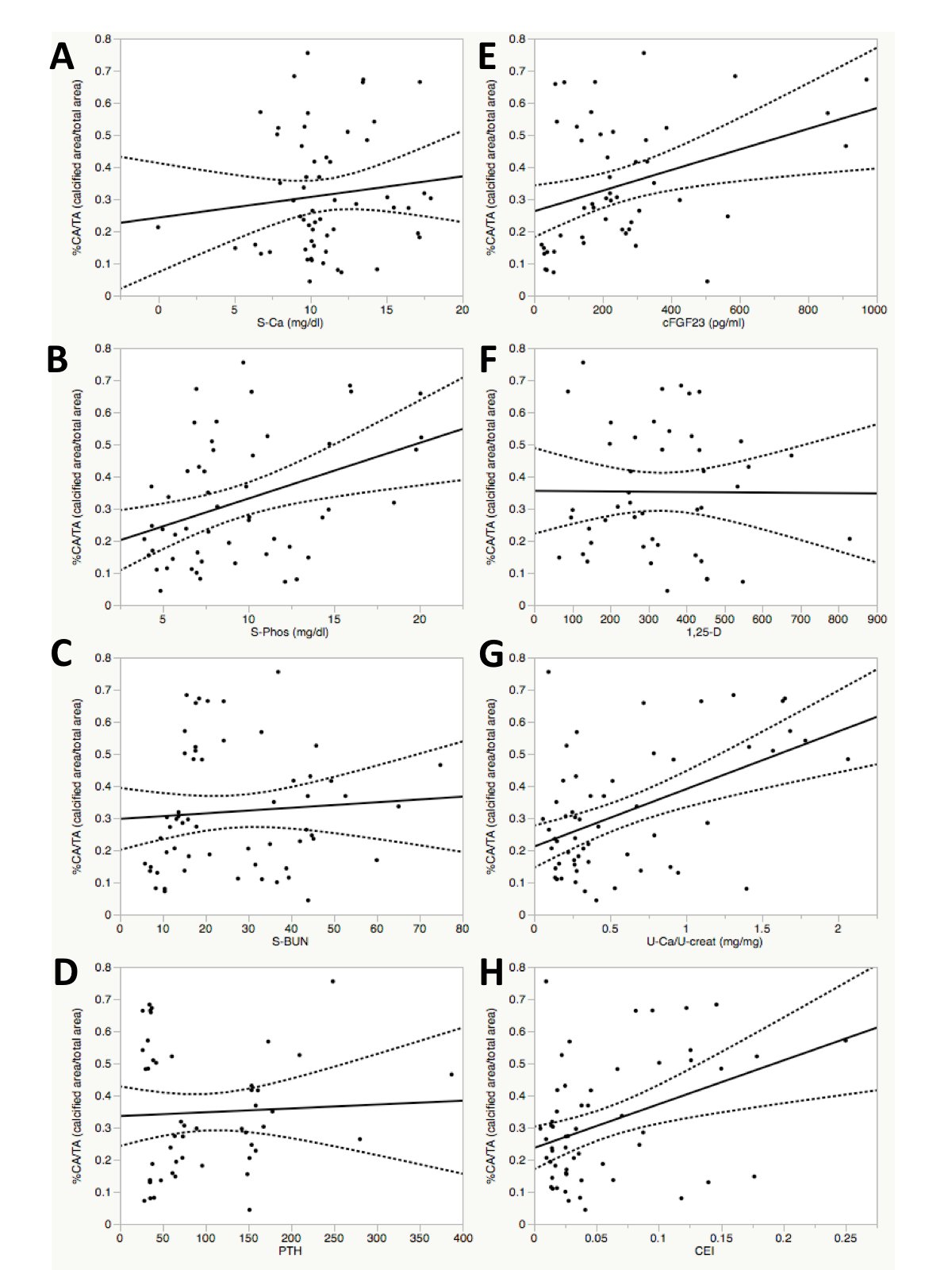

Supplement: S2 Fig — All experimental Npt2a-/- mice from S1 Table (n = 56) were evaluated using linear regression analysis to determine the association of renal mineralization with serum calcium (S-Ca, A), serum phosphorus (S-P, B), serum BUN (S-BUN, C), plasma intact PTH (PTH, D), plasma c-terminal FGF23 (cFGF23, E), serum 1,25(OH)2-vitamin D (1,25(OH)2-D, F), the ratios of urine calcium/urine creatinine (U-Ca/U-crea, G), and urine calcium excretion index (CEI, H). Data points represent values of individual animals. Results of the linear regression analysis are shown as solid line with 95% confidence interval (stippled lines), for correlation coefficients and Pearson’s p-values see Table 2. (TIFF) [file pone.0176232.s002.tiff]

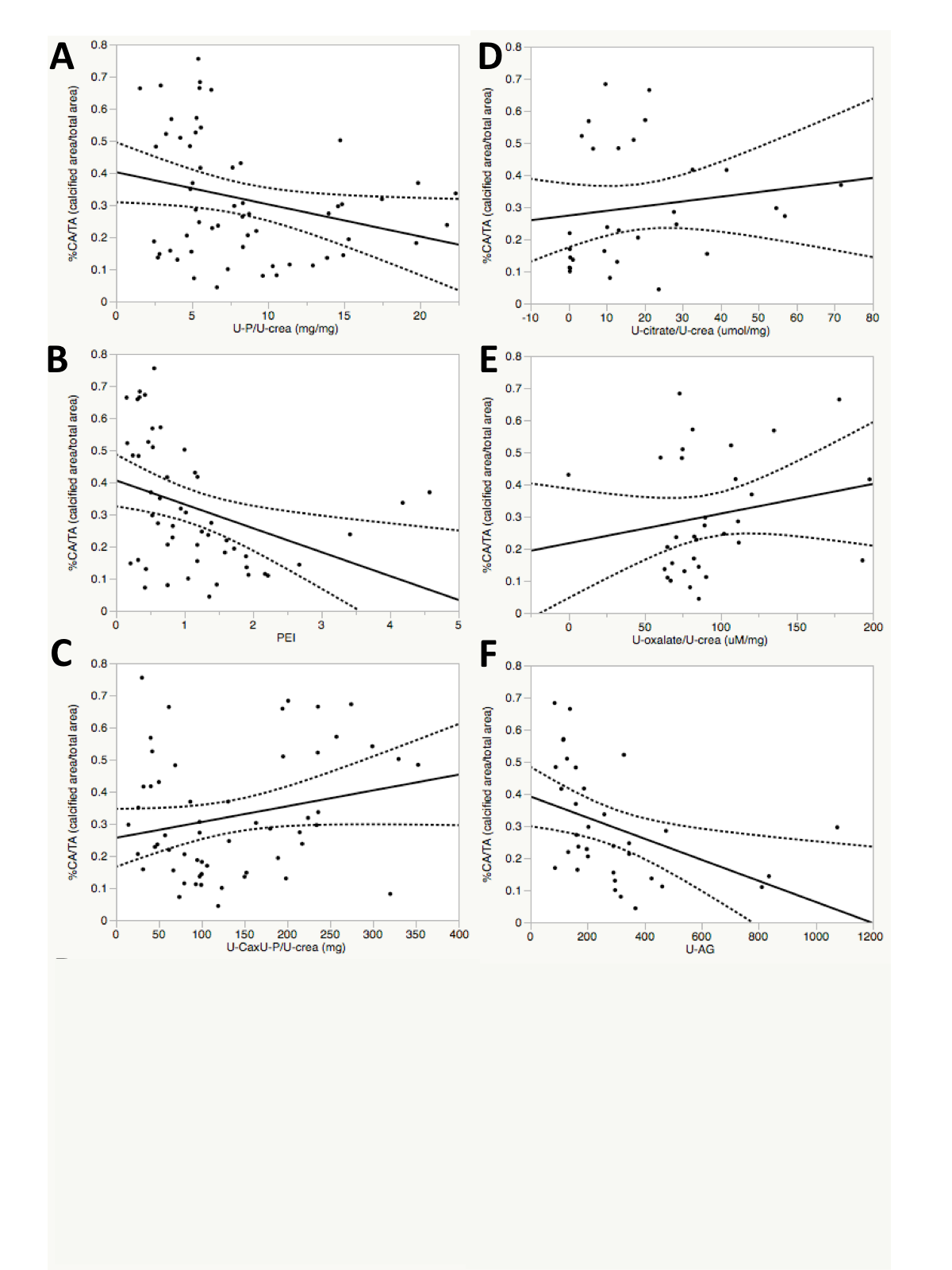

Supplement: S3 Fig — All experimental Npt2a-/- mice from S1 Table (n = 56) were evaluated using linear regression analysis to determine the association of renal mineralization with the ratios of urine phosphorus/urine creatinine (U-P/U-crea, A), urine phosphate excretion index (PEI, B), urine calcium*phosphorus/urine creatinine (U-Ca*U-P/U-crea, C), urine citrate/urine creatinine (U-citrate/U-crea, D), urine oxalate/urine creatinine (U-oxalate/U-crea, E), urine anion gap (U-AG, F). Data points represent values of individual animals. Results of the linear regression analysis are shown as solid line with 95% confidence interval (stippled lines), for correlation coefficients and Pearson’s p-values see Table 2. (TIFF) [file pone.0176232.s003.tiff]
